# Synthesis and Characterization of Lignin-Silver Nanoparticles

**DOI:** 10.3390/molecules29102360

**Published:** 2024-05-16

**Authors:** Dominik Maršík, Petter Paulsen Thoresen, Olga Maťátková, Jan Masák, Pavel Sialini, Ulrika Rova, Vasiliki Tsikourkitoudi, Paul Christakopoulos, Leonidas Matsakas, Irena Jarošová Kolouchová

**Affiliations:** 1 Department of Biotechnology, University of Chemistry and Technology, 166 28 Prague, Czech Republic; dominik.marsik@vscht.cz (D.M.); olga.matatkova@vscht.cz (O.M.); jan.masak@vscht.cz (J.M.); 2 Biochemical Process Engineering, Division of Chemical Engineering, Department of Civil, Environmental and Natural Resources, Luleå University of Technology, 971 87 Luleå, Sweden; petter.paulsen.thoresen@ltu.se (P.P.T.); ulrika.rova@ltu.se (U.R.); paul.christakopoulos@ltu.se (P.C.); 3 Central Laboratories, University of Chemistry and Technology, 166 28 Prague, Czech Republic; pavel.sialini@vscht.cz; 4 Department of Microbiology, Tumor and Cell Biology, Karolinska Institutet, 171 77 Stockholm, Sweden; vasiliki.tsikourkitoudi@ki.se

**Keywords:** lignin-silver nanoparticles, green synthesis, plasmon resonance

## Abstract

Metal nanoparticle synthesis via environmentally friendly methods is gaining interest for their potential advantages over conventional physico-chemical approaches. Herein, we propose a robust green synthesis route for lignin-modified silver nanoparticles, utilizing the recovery of lignin as a renewable raw material and exploring its application in valuable areas. Through a systematic approach combining UV-Vis spectroscopy with AAS and DLS, we identified repeatable and scalable reaction conditions in an aqueous solution at pH 11 for homogeneous silver nanoparticles with high uniformity. The TEM median sizes ranged from 12 to 15 nm with circularity between 0.985 and 0.993. The silver nanoparticles yield exceeded 0.010 mol L^−1^, comparable with traditional physico-chemical methods, with a minimal loss of silver precursor ranging between 0.5 and 3.9%. Characterization by XRD and XPS revealed the presence of Ag-O bonding involving lignin functional groups on the pure face-centered cubic structure of metallic silver. Moreover, the lignin-modified silver nanoparticles generated a localized thermal effect upon near-infrared laser irradiation (808 nm), potentially allowing for targeted applications in the biomedical field. Our study showcases the potential of lignin as a renewable reducing and capping agent for silver nanoparticle synthesis, addressing some shortcomings of green synthesis approaches and contributing to the development of suitable nanomaterials.

## 1. Introduction

Currently, the synthesis of metal nanoparticles using environmentally friendly methods is gaining prominence, offering both advantages and disadvantages compared with conventional physico-chemical methods. These green approaches boast adherence to sustainable chemistry principles, often involving straightforward synthesis processes and leveraging the beneficial properties conferred by intricate functional compounds present on the surface of a metal core. Such compounds can enhance stability and impart desired characteristics to the nanoparticles [1]. However, these methods face limitations when it comes to scaling up production, primarily due to issues related to yield, material availability, variability, and the challenge of achieving consistent synthesis control and repeatability [2]. Despite these challenges, lignin (Lig) emerges as a promising raw material due to its widespread availability and continuous exploration for valuable applications, although a majority of lignin is currently incinerated for energy generation postprocessing [3,4,5], primarily due to its resilient and intricately cross-linked phenylpropanoid structure [6]. On the other hand, silver nanoparticles (AgNPs) represent the most extensively utilized nanomaterial in commercial applications, with their usage continually expanding [7].

In introducing the synthesis of Lig-AgNPs, we apply the established physico-chemical characteristics of AgNPs. Notably, AgNPs exhibit light–matter interactions due to collective plasmon oscillations of conduction electrons on the metal surface when irradiated with light at their plasmon frequency [8,9]. These interactions, known as localized surface plasmons (LSPs), play a crucial role in determining the optical properties of AgNPs within the size range of approximately 10 to 100 nm. In this extrinsic regime, the resonance frequency is highly sensitive to factors such as NP size, shape [10], agglomeration state, surface modification, dielectric properties, and the refractive index of the surrounding medium [11]. For small spherical NPs (up to ~20 nm), light absorption is dominant, with the peak intensity occurring around 400 nm. The broadening and redshifting of the peak indicate the growth in the size of the AgNPs [12], which leads to an increase in the scattering contribution to the extinction coefficient. On the other hand, an increase in nanoparticle size while maintaining a constant mass concentration of silver results in a decrease in the number of NPs in the sample. In the case of these two opposing phenomena, the decrease in the number of particles in the sample gradually prevails with the increasing diameter of NPs, which results in a decrease in the absorbance value [13].

Scattering during NPs aggregation has a similar effect on extinction. Upon aggregation, the conduction electrons on the surface of the NPs are shared among neighboring particles, resulting in a decrease in the absorbance value due to a lower number of stable NPs. This is often associated with peak broadening or the appearance of a second peak at longer wavelengths [14,15]. Taking into consideration the nucleation mechanism, the size or susceptibility to aggregation of AgNPs can be influenced by the input concentration of reagents. Takesue et al. [16] observed three phases of NPs formation during the reduction of AgNO_3_ using a reducing solution composed of sodium citrate and tannic acid followed by adjusting the pH to 12. The process involved the reduction of Ag^+^ ions to Ag^0^, leading to the formation of Ag_13_ clusters during the induction phase. These clusters then nucleate and are consumed in the formation of AgNPs. The final phase involves the growth of smaller NPs through coalescence and aggregation to form larger NPs. Instead of tannic acid, among many classes of reductors described elsewhere [17], lignin can be applied, additionally ensuring the capping and stabilization of the silver core [18]. To induce the reduction of the precursor in the form of silver salt to Ag^0^ using lignin, it is necessary to increase reaction temperature [18] and/or add a base [19]. It is suggested that the reduction can take place by two different mechanisms depending on the pH. Under alkali conditions, the reduction of Ag^+^ is mediated autocatalytically on the surfaces of Ag_2_O. Under neutral and acidic conditions, direct reductions of Ag^+^ to Ag^0^ probably occurs differently due to the absence of Ag_2_O resulting to polydisperse NPs [20]. The reduction of the precursor by lignin is ensured by aliphatic hydroxyls, phenolic hydroxyls, and thiols normally present in the polymer structure. Functional groups of primary and secondary alcohols and aldehydes are known as sacrificial oxidation agents and induce H^+^ abstraction under alkaline conditions. NaOH accelerates the self-oxidation of lignin by reducing formed H^+^ and increasing the reducing speed [21], which supports system homogeneity [22]. Additionally, the presence of polar sulfonate groups supports NP dispersion in aqueous solution [18].

In the context of AgNPs’ morphology and its consequential impact on optical properties, it becomes imperative to account for the influence of temperature during synthesis. Gonzáles et al. [23] demonstrated that at room temperature, colloidal solutions of AgNPs smaller than 30 nm predominantly form faceted near-spherical shapes with similar optical emissions. However, with increasing temperature and/or size NPs (40 nm and above), different morphologies can be observed in experimental samples, leading to changes in the optical properties, including shifts in the maximum absorption wavelength or alterations in absorbance values while maintaining the mass concentration of silver [24].

The optical properties of NPs can also be tuned by external influences such as the dielectric properties and refractive index of the external medium. Generally, the extinction is redshifted as the refractive index increases, whether they are in polar or non-polar solvents [11]. However, Serra et al. [25] found no change in the optical characteristics after adding water to an ethanol solution of the spherical AgNPs. Conversely, Vodnik et al. [26] observed a redshift caused by a change in the dielectric constant of the surrounding medium when transferring water-phase spherical silver NPs using oleylamine as the transfer/capping agent to chloroform, though the absorbance values remained unchanged [26]. The capping agent itself could also impact the optical properties and enhanced the absorption of AgNPs through its dielectric properties [27]. Lignin, with its complex structure containing conjugated bonds with high electron polarizability and a lower energy threshold for absorption and electron transitions, significantly contributes to its dielectric function. The extinction coefficient of softwood kraft lignin increases at wavelengths below 700 nm due to absorption in the lower UV region [28].

Taking into consideration these aspects, our study aimed to develop a reliable and straightforward method for synthesizing lignin-modified silver nanoparticles (Lig-AgNPs) with high yield and uniform properties. Applying the insights gained from the mechanism of AgNPs formation and their optical properties, we conducted a comprehensive series of experiments covering a wide range of lignin input concentrations and varying ratios between lignin and silver precursors. Utilizing UV-Vis (ultraviolet-visible) spectroscopy, an accessible and simple technique, facilitated this process. Upon identifying suitable conditions, we progressed to the scale-up phase, where we corroborated our findings using AAS (atomic absorption spectroscopy) and DLS (dynamic light scattering). These additional methods allowed for the validation and accuracy of the synthesis conditions determined based on UV-Vis spectroscopy. Subsequently, we conducted a further scale-up process. Finally, employing techniques such as FTIR (Fourier-transform infrared spectroscopy), XPS (X-ray photoelectron spectroscopy), and XRD (X-ray diffraction) alongside TEM (transmission electron microscopy) allow for the comprehensive characterization of both the envelope and core of the Lig-AgNPs. This characterization provides valuable insights for their potential utilization in various industrial applications.

## 2. Results and Discussion

### 2.1. Lignin Solubility

In order to prepare the Lig-AgNPs, the lignin needs to be dissolved in an aqueous solution. Lignin is generally insoluble in water, and to solubilize lignin, the pH was increased by the gradual addition of NaOH to reach pH values of 10.00, 11.00, and 12.00. As shown in Table 1, lignin solubility increased with higher pH; however, at pH 10.00 and pH 11.00, the solubility gradually decreased upon the increase of lignin initial concentration. Alternatively, to achieve enhanced solubility, a predetermined quantity of lignin was combined with a 60% (*v*/*v*) aqueous ethanol solution, leading to an almost 100% solubility at all tested pH values. Previous studies by Hu and Hsieh [20] explored the synthesis of Lig-AgNPs by using alkali lignin in the pH range of 5.98 to 10.01, observing an increasing absorbance band centered between 411 and 421 nm with increasing pH. Our preliminary tests of Lig-AgNPs synthesis showed an increasing trend of absorbance, even above a pH value of 10.01. To ensure comparable synthesis conditions in both water and 60% (*v*/*v*) aqueous ethanol solution, a pH value of 11.00 was finally chosen. A pH value of 12.00 was evaluated as disadvantageous due to challenges in stabilizing the pH during the entire experiment and the total NaOH consumption. Our selected lignin solubility conditions represent an optimal balance, achieving nearly 100% lignin solubility while utilizing moderate pH values, addressing economic considerations, and minimizing environmental impact. A gradual dissolution process is designed to establish reproducible conditions for the synthesis of Lig-AgNPs.

### 2.2. Approptiate Conditions for the Preparation of Lig-AgNPs

To indicate the formation of Lig-AgNPs, we exploited the light absorption and scattering properties of AgNPs (AgNPs). For this purpose, we examined the absorption spectra in the range of 300–800 nm to find suitable initial concentrations of lignin and the mass reaction ratio between silver ions and lignin. We conducted the experiment in two different solvents, ultrapure water, and a 60% aqueous ethanol solution (*v*/*v*). The evaluation of the maximum absorbance A_MAX(400–800) nm_ for individual spectra was performed within the range of 400–800 nm due to the partial overlap of the lignin absorption spectrum (Figure S1) with the typical absorption of spherical AgNPs, which occurs between 400 and 500 nm [29].

**Figure 1:**
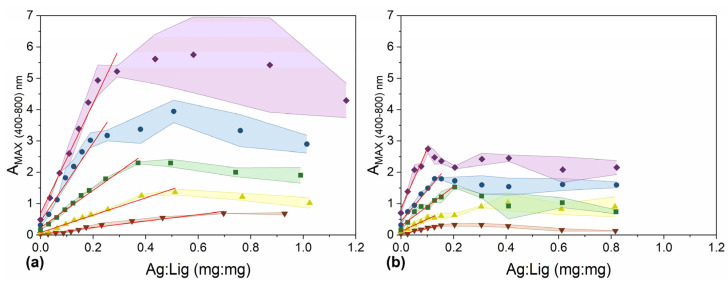
The maximum absorbance value ranging from 400 nm to 800 nm of unseparated (P+S) Lig-AgNPs on the reaction mass ratio between Ag^+^ and lignin at different initial lignin concentrations, 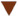 1.00 g L^−1^, 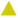 2.50 g L^−1^, 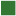 5.00 g L^−1^, 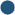 10.00 g L^−1^, and 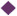 20.00 g L^−1^, in a final volume of 210 µL. (**a**) Water, pH 11.00; (**b**) 60% (*v*/*v*) aqueous ethanol solution, pH 11. The red lines are the results of a linear regression and include points with R-square (COD) ≥ 0.95. Colored areas represent standard deviation values of 3 independently prepared batches of Lig-AgNPs.

From the absorption spectra obtained after the nanoparticle preparation in a total volume of 210 µL in the microtiter plate, we plotted the dependence of A_MAX(400–800) nm_ on the corresponding reaction ratio (Figure 1). The slight shift on the *X*-axis between individual lignin initial concentrations is caused by the different concentration of lignin (Table 1). Linear regression analysis was performed for each curve to ensure that the R-square (COD) was ≥0.95 (Table 2). We infer that when the absorbance decreases to a point where the curve no longer aligns with the linear regression, it indicates a notable deviation from the desired homogeneity of the synthesized NPs. This deviation may imply undesirable alterations, such as the formation of aggregates, an overall enlargement in the mean size of NPs, variations in size distribution, and/or a reduction in the yield of AgNPs [10,11,12,13,14,15].

Comparing the linear regression parameters (Table 2) obtained for reactions in water, we noticed a proportional increase in the slope depending on the initial concentration of lignin of up to 10.0 g L^−1^. Within the range of silver ions and lignin mass reaction ratios that exhibit linearity for lignin initial concentrations of 1.0 g L^−1^ to 10.0 g L^−1^, we hypothesize that the properties of the nanoparticle system as a whole will remain similar. The wide reaction range was likely due to the rapid reduction and nucleation facilitated by the addition of NaOH [21]. The nearly identical nucleation time allowed most crystals to grow to nearly identical sizes, as the particles experienced the same growth conditions and history [30]. The fast reaction rate of Ag^+^ results in a high supersaturation S [21], a critical parameter influencing the induction time [31], defined as the natural logarithm of the ratio of AgNO_3_ concentration C to the saturation concentration of silver in solution $C^{*}$ = 2 ∙ 10^−12^ mol L^−1^ [32,33]. In our experiment layout, the supersaturation range was S = 18.5 (initial concentration 1.0 g L^−1^, reaction ratio 0.03) and  S = 22.9 (initial concentration 10.0 g L^−1^, reaction ratio 0.25), and no significant difference in crystal growth was observed that led to a change in the mean size of Lig-AgNPs. A decrease in the slope value at the initial concentration of 20.0 g L^−1^ could be attributed to the high ionic strength of the solution, which may lead to particle coalescence [34]. The apparent gradual decrease in reaction ratios that met R-square (COD) ≥ 0.95 with increasing lignin concentration (Figure 1a) may be ascribed to the increasing reagent concentrations. The input concentration of reagents affects the nucleation process, during which molecular clusters form the new crystalline phase [35]. Consequently, with increasing concentration, more frequent collisions between particles occur, leading to the formation of lumps or aggregates that cannot subsequently absorb light as efficiently as individual NPs [15,36,37,38].

**Scheme 1:**
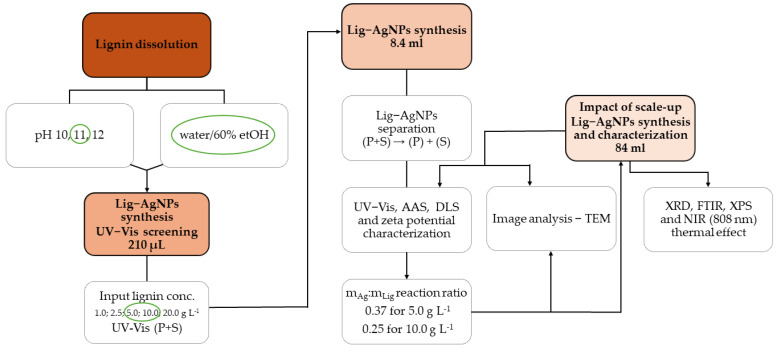
Exploration of suitable conditions for Lig-AgNPs synthesis and characterization techniques. P+S represent Lig-AgNPs characterized without subsequent separation, P represents the separated Lig-AgNPs, S represents the supernatant depleted of Lig-AgNPs. The green circles show the selected conditions based on the experiments performed.

We selected a lignin concentration of 5.0 g L^−1^ and 10.0 g L^−1^ for the scale-up phase in water (Scheme 1). The 5.0 g L^−1^ input concentration represents a compromise between a sufficiently high yield of Lig-AgNPs and the utilization of lignin, as the linearity trend was maintained up to a ratio of 0.37. The 10.0 g L^−1^ input concentration was chosen for the higher yield in the same volume compared with the series 5.0 g L^−1^, 2.5 g L^−1^, and 1.0 g L^−1^ (Figure 1a). The highest initial concentration of 20.0 g L^−1^ was excluded due to a decrease in the slope value of the line (Table 2).

To explore an alternative synthesis process for achieving complete solubility of lignin, the reactions were conducted in 60% ethanol with a pH of 11. Due to the almost complete solubility of lignin under these conditions, the values of the ratios are lower compared with the water (Figure 1). It is essential to note that ethanol, being a second reducing agent, not only impacts the solubility of lignin but also influences the silver nanoparticle formation process. In our experimental setup, the formation of Lig-AgNPs was not observed when synthesis was performed using lignin dissolved in a 60% ethanol solution (pH~4.20) without the addition of NaOH, as analyzed by UV-Vis spectroscopy. This finding aligns with the observations by Cai et al. [39], where they found that Ag^+^ and more easily reducible Ag_2_O were not reduced in pure ethanol. However, upon the addition of a base, Ag^+^ ions were converted to Ag_2_O, which could be further reduced by ethanol in the presence of residual hydroxyl ions. Hence, both reducing agents (lignin and ethanol) require the presence of a base for the synthesis of AgNPs under standard conditions. It is known that the pH needed to convert Ag^+^ to Ag_2_O exceeds 10.5 [40], suggesting that Lig-AgNPs are apparently synthesized through the autocatalytic reduction of Ag^+^ via Ag_2_O intermediates, as proposed in the study by Hu and Hsieh [20].

When comparing the synthesis performed in water and in 60% ethanol solution, we can observe that the curves following the linear trend generally have fewer data points (Figure 1). In other words, the similar properties of Lig-AgNPs observed in water were not preserved over such a wide range of initial Ag^+^ concentrations in the 60% ethanol solution. The higher solubility of lignin in the ethanol-based reaction medium leads to a large number of lignin molecules present in the mixture that are capable of participating in the Ag^+^/Ag_2_O reduction compared with water, making depletion of the lignin responsible for the reduction unlikely at these low reaction ratios. The explanation could be the lower concentration of NaOH that is required to achieve a stable pH of 11 in the 60% ethanol solution compared with water. Consequently, the reduction activity of lignin is likely decreased because the formation of H^+^ associated with lignin and ethanol oxidation lowers the pH, thereby affecting the standard reduction potential [21]. Next, the lower absorbance values observed in reaction ratios containing the same initial amount of Ag^+^ compared with reactions conducted in water could be attributed to the presence of both ethanol and water during the synthesis of Lig-AgNPs. NaOH and AgNO_3_/Ag_2_O are more soluble in water than in ethanol, while lignin is more soluble in ethanol than in water. As a result, the reaction carried out in a mixture of water and ethanol is slower than in water alone, as the contact between the reagents is inhibited [39,41,42]. This slower reaction rate leads to a different growth history of the particles, resulting in a broader size distribution [22]. Additionally, the preferential reaction between Ag^+^ and NaOH leading to Ag_2_O can decrease the concentration of residual OH^−^ ions, which are essential for the autocatalytic reduction mechanism of Ag^+^. Considering these factors and the simplicity of the synthesis process, we have decided to perform the scale-up only in the context of water. Our methodology enables us to achieve streamlined synthesis conditions without the need for Lig-AgNPs separation, all within the context of microscale reactions. Furthermore, our findings demonstrate the elimination of organic solvent in Lig-AgNPs preparation, aligning with environmentally sustainable and economically viable AgNps synthesis.

**Figure 2:**
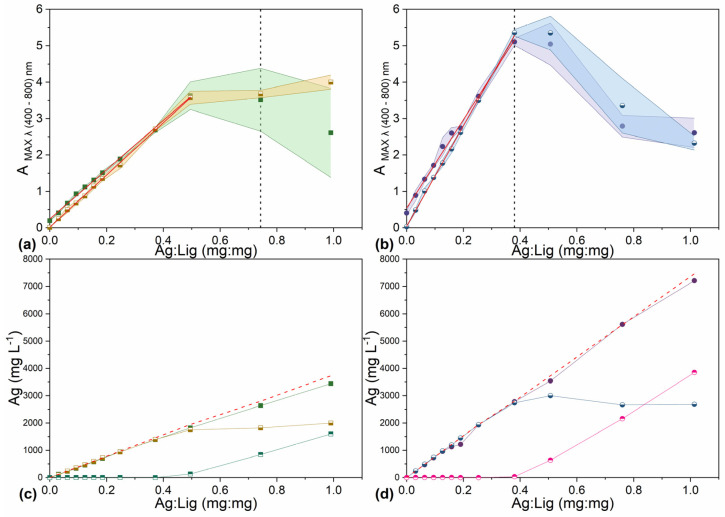
(**a**,**b**) The maximum absorbance value ranging from 400 nm to 800 nm of 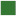

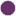
 unseparated (P+S) and 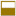

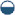
 separated (P) Lig-AgNPs on the reaction mass ratio between Ag^+^ and lignin in final volume of 8.4 mL. (**a**) Water, pH 11.00, initial lignin concentration 5.0 g L^−1^; (**b**) water, pH 11.00, initial lignin concentration 10.0 g L^−1^. The red lines are the results of linear regression and include points with R-square (COD) ≥ 0.95. Colored areas represent standard deviation values of 3 independently prepared batches of Lig-AgNPs. Dashed lines represent the point where visually observed aggregates form. (**c**,**d**) The mass concentration of Ag^+^ in 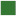

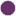
 unseparated (P+S) and 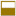

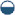
 separated (P) Lig-AgNPs and in 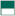

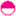
 supernatant (S). (**c**) Water, pH 11.00, initial lignin concentration 5.0 g L^−1^; (**d**) water, pH 11.00, initial lignin concentration 10.0 g L^−1^. The red dashed lines represent the calculated maximum mass concentration of Ag^+^.

For reactions carried out in a total volume of 8.4 mL, a higher number of reaction ratio values met the linear trend (Figure 2a,b) compared with reactions performed in a 40 times smaller volume (Figure 1a). This observation might be attributed to the different efficiency of homogenization between the microtitrate plate and the glass vessel [43]. Additionally, in the case of microtitrate plates, complete mixing was not ensured throughout the entire reaction duration, as mixing commenced after all wells were filled. When comparing the corresponding straight lines of unseparated (P+S) and separated (P) Lig-AgNPs (Figure 2a,b), a slight difference in absorbance was observed, which decreased as the reaction ratio increased. This reduction in absorbance in the initial ratios of P compared with P+S resulted from a partial overlap of the absorption spectra between lignin and the maximum absorbance for Lig-AgNPs (Figure S1). As the amount of Ag^+^ in the reaction mixture increased, so did the consumption of lignin, which remained constant within the initial concentration for the stabilization and capping of Lig-AgNPs. Consequently, the slope value of the P line increased compared with P+S (Table 3), leading to the equalization of the absorbance difference at the initial concentration of 5.0 g L^−1^ (Figure 2a) and 10.0 g L^−1^ (Figure 2b) in ratios 0.50 and 0.38, respectively. The increase in the slope value of the P line was a result of a higher amount of unreacted lignin in the mixture of the unseparated sample (P+S) at the initial reaction ratios. The efficiency of the separation process and the conversion of Ag^+^ to Lig-AgNPs were verified using AAS (Figure 2c,d). The maximum conversion was achieved at a lignin initial concentration of 5.0 g L^−1^ up to ratio 0.37, with the amount of Ag^+^ in the supernatant after the separation of the NPs ranging from 1.8 to 5.8 mg L^−1^. In the 0.50 ratio, which still followed a linear trend in UV-Vis determination, we observed an increase in Ag^+^ in the supernatant up to 132.0 mg L^−1^. Similarly, with an initial concentration of lignin at 10 g L^−1^, the maximum conversion of Ag^+^ was achieved up to the ratio of 0.25, with the Ag^+^ in the supernatant being in the range of 2.1–5.9 mg L^−1^. In the 0.38 ratio, which also still followed a linear trend, an increase in Ag^+^ in the supernatant was detected up to a value of 34.3 mg L^−1^. Hence, if the resulting reaction ratios were selected based on UV-Vis spectroscopy, the reaction would only be performed with a slight excess of silver, indicating the suitability of this method as a simple screening tool for selecting initial concentrations of reactants. The concurrence of ratios with reduced absorbance values with higher deviations and the increase in Ag^+^ in the supernatant suggests that the linearity was affected not only by the formation of aggregates but also by the depletion of reducing groups in the reaction mixture. The visually observed formation of aggregates settling on the walls of the glass vessel during the reaction (Figure S2) is indicated by the dashed lines in Figure 2a,b. Because the sum of the mass concentration of Ag^+^ in the separated (P) Lig-AgNPs samples and the supernatant (S) corresponded to the mass concentration of Ag^+^ in the supernatant (P+S) samples (Figure 2c,d), the determination of AAS can be considered a suitable complementary method to UV-Vis spectroscopy. Slight deviations from the maximum theoretical concentration are probably caused by the properties of the analyzed samples, which are not a true solution but a colloid.

**Figure 3:**
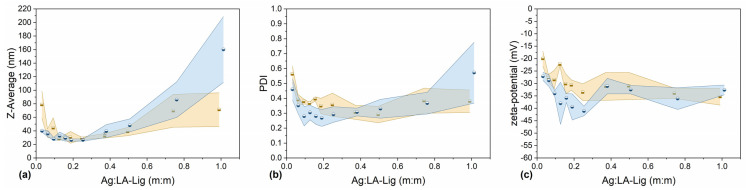
Effect of initial lignin concentration and mass reaction ratio between Ag^+^ and lignin on (**a**) hydrodynamic diameter, (**b**) polydispersity index, (**c**) zeta potential of separated (P) Lig-AgNPs 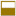 initial lignin concentration 5.0 g L^−1^; 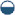 initial lignin concentration 10.0 g L^−1^. Colored areas represent standard deviation values of 3 independently prepared batches of Lig-AgNPs.

The determination of hydrodynamic diameter, PDI, and ζ potential served as complementary screening methods for characterizing the separated Lig-AgNPs prepared in all reaction ratios. Overall, all samples exhibited a strong negative charge ranging from −20 mV to −40 mV, indicating a moderate to highly stable colloidal stability of Lig-AgNPs (Figure 3c) [44]. The average size of Lig-AgNPs increased with increasing PDI values (Figure 3a,b). Based on this observation, we can infer that there is no proportional increase in the size of Lig-AgNPs with increasing Ag^+^ concentration. The larger size and higher polydispersity in the initial ratios might be related to the lower concentration of Ag^+^, which could alter the growth process, leading to NPs with a broader size distribution, including larger Lig-AgNPs, to which DLS is more sensitive. Upon increasing the addition of Ag^+^, Lig-AgNPs showed moderate polydispersity with a consistent hydrodynamic radius (Figure 3). In the highest ratios, a gradual increase in the average hydrodynamic size can be observed from 0.50 in the 5.0 g L^−1^ series and from 0.38 in the 10.0 g L^−1^ series (Figure 3a), which is consistent with ratios that still followed a linear trend after UV-Vis spectroscopy determination (Figure 2a and Figure 3b). Another increase in hydrodynamic diameter and PDI occurred due to aggregation during the synthesis (Figure S2), causing Lig-AgNPs to become highly polydisperse again.

**Figure 4:**
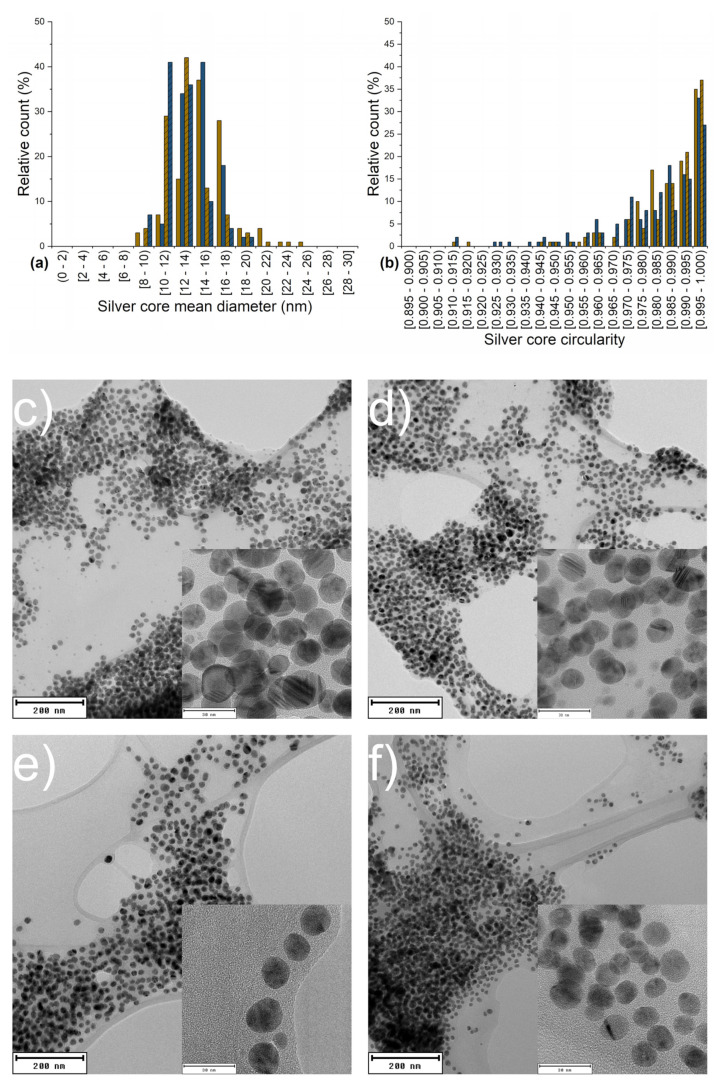
Effect of input lignin concentration, mass reaction ratios m_Ag_:m_Lig_, and reaction volume on (**a**) size distribution morphology and (**b**) circularity of the Lig-AgNPs core. TEM imaging with scales of 200 nm and 30 nm; 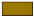 (**c**) 8.4 mL, 5.0 g L^−1^, 0.37 m_Ag+_: m_lig_; 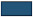 (**d**) 8.4 mL, 10.0 g L^−1^, 0.25 m_Ag+_: m_lig_; 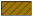 (**e**) 84.0 mL, 5.0 g L^−1^, 0.37 m_Ag+_: m_lig_; 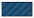 (**f**) 84.0 mL, 10.0 g L^−1^, 0.25 m_Ag+_: m_lig_.

To further scale up the reactions, we opted to perform them with a slight excess of lignin, specifically at the ratios where the maximum conversion of Ag^+^ to Lig-AgNPs was achieved. These ratios were 0.37 for the 5.0 g L^−1^ series and 0.25 for the 10.0 g L^−1^ series. The results of the screening methods for reactions conducted in a total volume of 84.0 mL are summarized in Table 4, which also includes the median size distribution of the Lig-AgNPs. An electron microscopy analysis revealed silver cores enveloped by lignin (Figure S3). The image analysis focused on the silver cores of Lig-AgNPs, displaying a narrow size distribution (Figure 4). The apparent difference in size between DLS and TEM dry sample measurements is attributed to various factors inherent to these techniques. DLS yields a larger hydrodynamic size due to the inclusion of the lignin envelope, possible aggregates, or impurities in the measurement. As a corrective measure, filtration is commonly recommended before DLS analysis to reduce the increased PDI [44]. This step was not included in our work as one of our aims was to find a reaction ratio where aggregation minimally occurs. Comparing the results obtained in lower volumes, we found no significant changes in system characteristics such as size, shape, optical properties, and homogeneity. Similarly, there were no significant differences observed when varying the initial concentration of lignin and the resulting reaction ratio. Nonetheless, a closer examination of silver cores in Lig-AgNPs through TEM imaging revealed a slightly narrower size distribution when prepared at an initial concentration of 10.0 g L^−1^ and a ratio of 0.25 (Figure 4a). The shape distribution of Lig-AgNPs appeared similar for all samples, and in general, the circularity increased with the relative count of Lig-AgNPs (Figure 4b). Additionally, more than 90% of silver cores exhibited a circularity value greater than 0.95, and no NPs with a circularity below 0.90 were observed.

Hence, we infer that the scale-up has no significant impact on the synthesis of Lig-AgNPs under consistent reaction conditions (Table 4). A review conducted by Li et al. [45] observed that common chemical methods yield stable colloids with Ag concentrations below 0.010 mol L^−1^, but above this concentration, they tend to become unstable and form aggregates. In our study, employing an initial lignin concentration of 5.0 g L^−1^ in a ratio of 0.37 m_Ag_:m_Lig_ resulted in a Ag concentration of approximately 0.013 mol L^−1^, and with an initial lignin concentration of 10.0 g L^−1^ in a ratio of 0.25 m_Ag_:m_Lig_, we attained around 0.018 mol L^−1^ of Ag. This indicates that a robust biosynthesis of lignin-modified AgNPs can be achieved, carried out under mild conditions, and they present a competitive alternative to common physico-chemical methods.

In summary, our chosen reaction conditions represent a novel and appropriate approach, combining a high loading of lignin solution with a substantial yield of Lig-AgNPs and maximal lignin utilization in an aqueous environment. Another notable advantage is the near-complete utilization of silver precursor, enhancing process efficiency. An additional benefit of our method is the narrow size distribution of Lig-AgNPs with a circularity exceeding 0.95. Importantly, these positive aspects are maintained during the scale-up of the entire process.

### 2.3. Core and Surface Characterisation of Lig-AgNPs

**Figure 5:**
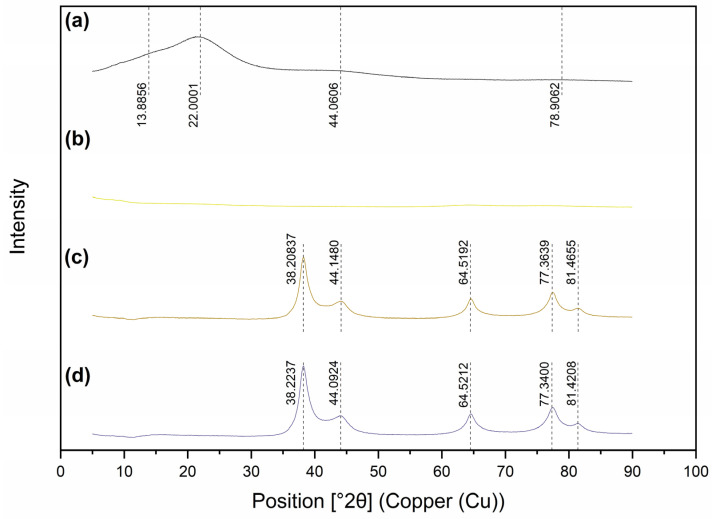
Powder X-ray diffraction patterns for (**a**) raw lignin; (**b**) lignin after NaOH treatment to pH 11; (**c**) separated (P) Lig-AgNPs synthesized in a total volume of 84.0 mL, water, pH 11.00, initial lignin concentration 5.0 g L^−1^, reaction ratio 0.37 m_Ag_:m_Lig_; (**d**) separated (P) Lig-AgNPs synthesized in a total volume of 84.0 mL, water, pH 11.00, initial lignin concentration 10.0 g L^−1^, reaction ratio 0.25 m_Ag_:m_Lig_.

To characterize the core of Lig-AgNPs, X-ray diffraction (XRD) analysis was employed (Figure 5) [46]. In both analyzed samples of Lig-AgNPs, the crystalline phase was identified as a pure face-centered cubic structure of metallic silver. The intensities conformity indicates that the crystallographic direction is not preferentially oriented. The crystallite size, determined using the Scherrer equation, was approximately 6 nm in both Lig-AgNPs samples. In comparison, the average diameter of the silver core in Lig-AgNPs, as determined by TEM image analysis, was 13 ± 2 nm with a lignin initial concentration of 5.0 g L^−1^ and 12 ± 2 with a lignin initial concentration of 10.0 g L^−1^. This suggests that a significant portion of the AgNPs in the analyzed samples are polycrystalline in nature [36]. The amorphous phase was analyzed in a sample of raw lignin and in lignin dissolved using the addition of sodium hydroxide to reach pH 11. Several dispersion bands can be seen in the amorphous pattern of raw lignin (Figure 5a). The values of interplanar distance (d-spacing) in the peak maxima align well with the parallel planes of polymer chains and match the d-spacing of the strongest diffraction maxima of cellulose. This is likely the result of cellulose molecules remaining in the lignin solution after the organosolv process [47]. From the diffraction pattern (Figure 5b), it is evident that after the pH 11 value adjustment, the structure of parallel polymer chains completely disappears [48]. The literature provides conflicting opinions regarding the exact interaction mechanisms of NaOH with cellulose and the mutual transition between individual cellulose I and cellulose II configurations [49]. Mercerization, or the exposure of cellulose to NaOH, induces swelling and the formation of Na-Cellulose, exhibiting distinct diffraction patterns [50]. Despite cellulose generally being poorly soluble in aqueous NaOH solutions [51,52], there are reports of cellulose with broken intramolecular hydrogen bonds being soluble in alkali solutions. Kamide et al. demonstrated complete solubility in a cellulose sample regenerated from an organic solvent solution through sulfuric acid precipitation, which is a process involving similar chemical agents to our organosolv process. In this instance, the amorphous content significantly increased after exposure to hydroxide solution, resulting in a reduction of intensity in the diffraction pattern [51]. Therefore, we suggest that residual cellulose could have been concurrently dissolved during our lignin dissolution process. This was then preserved during sample preparation (freezing and lyophilization) and analyzed in an amorphous phase, resulting in a reduction of signal in the diffraction pattern.

**Figure 6:**
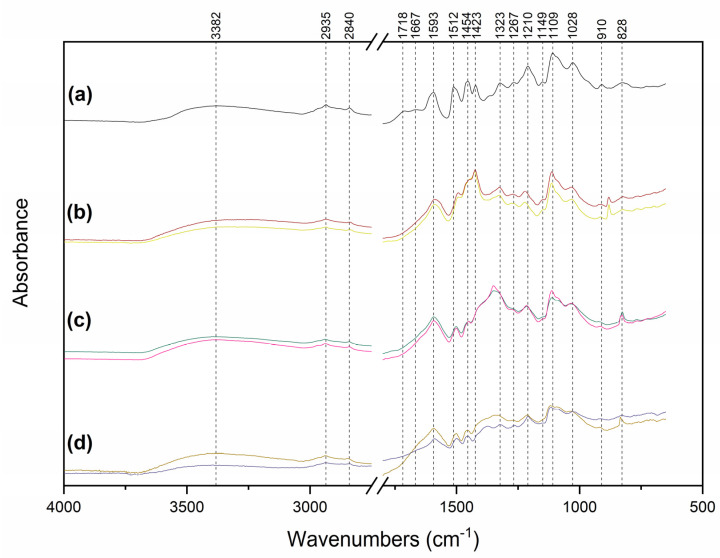
FTIR spectra of (**a**) 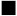 raw lignin; (**b**) lignin after NaOH treatment to pH 11, initial lignin concentrations 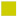 5.0 g L^−1^ and 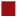 10.0 g L^−1^; (**c**) supernatant (S) after Lig-AgNPs separation, initial lignin concentrations 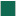 5.0 g L^−1^ and 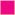 10.0 g L^−1^; (**d**) separated (P) Lig-AgNPs synthesized in a total volume of 84.0 mL, water, pH 11.00, 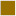 initial lignin concentration 5.0 g L^−1^ and reaction ratio 0.37 m_Ag_:m_Lig_ and 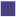 initial lignin concentration 10.0 g L^−1^ and reaction ratio 0.25 m_Ag_:m_Lig_.

In order to evaluate the lignin structures and their role during the formation of AgNPs, Fourier-transform infrared spectroscopy (FTIR) was performed (Figure 6) [53]. Several distinct bands are present in the raw lignin. First, the bands at 1109 and 1028 cm^−1^ have been previously attributed aromatic C-H in-plane deformations [54], where the higher wavenumber band in general is ascribed the syringyl unit and the latter the guaiacyl monomer. Furthermore, concerning the aromatic units, the bands at 1593 and 1512 cm^−1^ have been assigned C=C and C-C skeletal vibrations [55,56]. As with the bands occurring for wavenumbers 1109 and 1028 cm^−1^, the bands at 1454 and 1423 cm^−1^ have been attributed aromatic skeletal vibrations and C-H deformations [54]. Whereas the aromatic nature of the materials is unequivocal, there are certain variations among the various samples, which deserve some attention.

First are the bands present at 1423 and 1210 cm^−1^, which appear to interchange in intensity when comparing the raw lignin with that isolated subsequent dissolution under alkaline conditions. Suitably, the band at 1210 cm^−1^ has also been assigned C-O stretching [56], whereas the band close to 1423 cm^−1^ has been attributed phenolic hydroxyls [57]. Another point that requires attention is the signal emerging between wavenumbers 1423 and 1323 cm^−1^ for the supernatant (S) and pellet (P). For the pellet, the 5.0 g L^−1^ sample displays a greater absorption for hydroxyl groups (O-H stretching; 3382 cm^−1^) in addition to a large and smothered band between 1423 and 1323 cm^−1^. Knowing that aromatic O-H signals have been reported at intermediate wavenumbers (1397 cm^−1^) [57], the smothering could originate from a varied level of hydration of these specific groups, as has been reported to occur for hydrated carbonyls [58]. Still evaluating the pellet samples, lower associated moisture, as visualized by the band for wavenumbers 3700–3000 cm^−1^ [54], decrease the band associated with C=C skeletal vibrations [55,56], whereas it increases the out-of-plane C-H vibrations in the lower wavenumber range (1000–750 cm^−1^) [54]. The same is not observed for the previously dissolved lignins, where instead, higher associated moisture increase aromatic C-H deformation (in- and out-of-plane) [54]. Meanwhile, this instead appears to be a result from concentration and is thus more likely related to lignin assembly in the dissolved state, which will be discussed further.

For the supernatant lignins, this does not occur, and there are generally no differences between the two spectra. The size of the O-H bands is worth mentioning further, as lignin in the presence of moisture is reported to favor a spherical conformation by assembling hydrophobic and hydrophobic domains appropriately [59]. When evaluating the findings of the latter reference, this would correspond to the water hydration of polar groups and the hydrophobic stacking of aromatic motifs. Intuitively, a tightly packed lignin conformer would make it less likely for energy absorption through C-H out-of-plane deformations; meanwhile, a delocalized, stacked assembly of aromatic π-systems would absorb light efficiently, thus giving rise to the prominent C=C skeletal band favored by water hydration. Considering that lignin oxidation is the driver behind silver nanoparticle formation, its ability to act as an electron donor is naturally important and related to the lignin assembly’s ability to remain stable in an oxidized form [60,61]. Considering the formation of AgNPs through electron donation, and presuming that the lignin assembly’s zeta potential is indicative of the drive towards electron donation—which is reasonable as oxygen functional groups are reported important [62]—as a general rule of thumb, zeta potential increases decreases with particle size [63], whereas particle size increases with initial lignin concentration [64]. A compact lignin conformation should thus be more efficient for the formation of silver nanoparticles. Furthermore, this should be visible through lower absorbance of bands in the FTIR spectra from aromatic C-H out-of-plane deformations, whereas C=C skeletal vibrations should display elevated absorption as indicative of a more tightly assembled lignin [65,66]. This is observed for the separated Lig-AgNPs (P) when applying a higher silver to lignin ratio, indicating that more of the lignin is required to assemble to stabilize its oxidized state. An interesting notion in this sense is the associated absorption band from water, which could additionally serve to stabilize the lignin in this packed conformation. Previous reports on lignin oxidation and its role on silver ion reduction and the formation of AgNPs, especially carboxyl, carbonyl, and phenolic and hydroxylic groups, have been highlighted [62]. This is not in contrast to what has been found here, but in addition, lignin packing and ability to stabilize an oxidized state is important and is illustrated by the low absorption of out-of-plane C-H groups and instead the high absorption of C=C skeletal vibrations, indicative of an efficiently delocalized system, which can remain stable upon electron donation.

Finally, and worth mentioning in light of the previous discussion, are the methoxy groups in lignin. The two bands around 2935 and 2840 cm^−1^ have been assigned to C-H and CH_2_ stretching [54,67]. The band at 2935 cm^−1^ is usually assigned to methoxy C-H stretches. For the methoxyl C-H bends, the bands are usually located around 1460 cm^−1^ (asymmetric) and 1365 cm^−1^ (symmetric) [68]. Meanwhile, the symmetric methoxy C-H is reportedly not included in this band [54] and is thus likely instead to originate from aromatic O-H with varying degrees of hydration.

**Figure 7:**
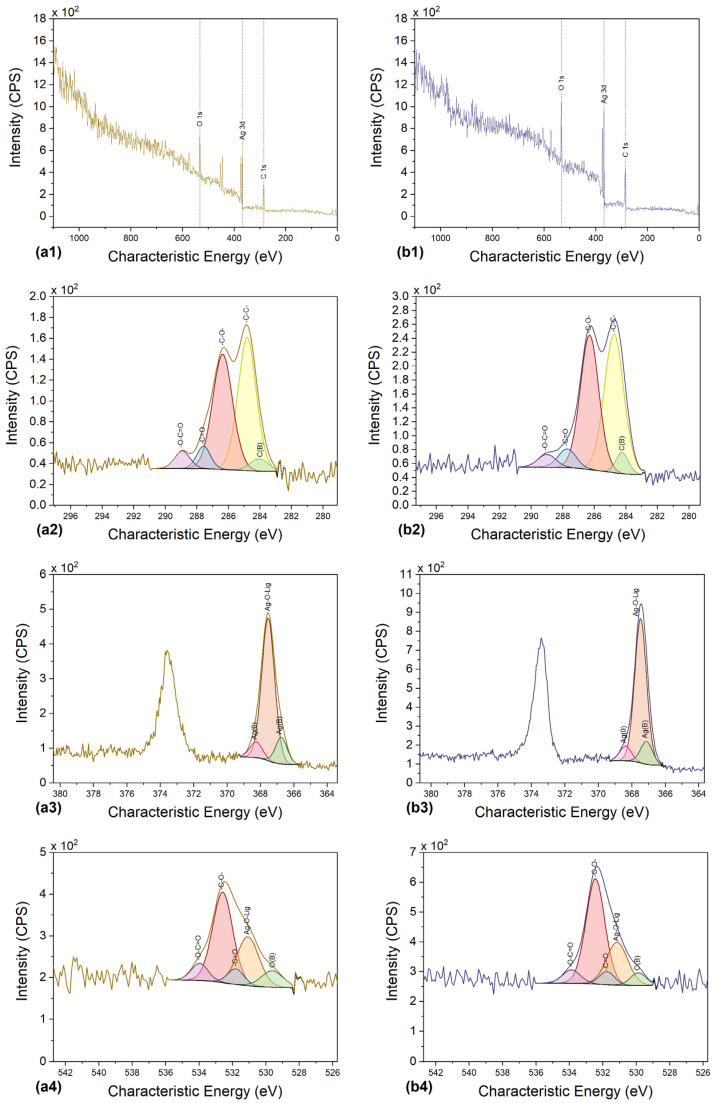
XPS spectra of separated (P) Lig-AgNPs synthesized in a total volume of 84.0 mL, water, pH 11.00 (**a1**) 5.0 g L^−1^, 0.37 m_Ag_:m_Lig_, wide scan; (**b1**) 10.0 g L^−1^, 0.25 m_Ag_:m_Lig_, wide scan; (**a2**) 5.0 g L^−1^, 0.37 m_Ag_:m_Lig_, C1s; (**b2**) 10.0 g L^−1^, 0.25 m_Ag_:m_Lig_, C1s; (**a3**) 5.0 g L^−1^, 0.37 m_Ag_:m_Lig_, O1s; (**b3**) 10.0 g L^−1^, 0.25 m_Ag_:m_Lig_, O1s; (**a4**) 5.0 g L^−1^, 0.37 m_Ag_:m_Lig_, Ag 3d; (**b4**) 10.0 g L^−1^, 0.25 m_Ag_:m_Lig_, Ag 3d.

To characterize the elemental composition of separated Lig-AgNPs, a wide scan of the samples using Ag 3d, C 1s, and O 1s orbitals in X-ray photoelectron spectroscopy (XPS) was performed (Figure 7). In the C 1s orbital, two dominant components were observed: C-C with sp2, sp3 hybridization (284.8 ± 0.4 eV) and C-O (286.3 ± 0.4 eV). Other functional groups, namely C=O (287.8 ± 0.4 eV) and O-C=O (289.0 ± 0.4 eV), were also detected, although in lower abundance. On the O 1s orbital, two major components were identified: C-O (532.6 ± 0.4) and Ag-O-lignin (531.0 ± 0.4 eV). The shift to a higher value of Ag-O-lignin binding energy compared with Ag-O (typically 530.0 eV) likely results from the presence of lignin. This shift in the oxygen binding energy is commonly observed in other organometallic compounds with oxygen-mediated binding [69]. Additionally, the functional groups =O (531.4 ± 0.4) and O-C=O (533.6 ± 0.4) were detected on the O 1s orbital. On the Ag 3d orbital, the main component observed for all samples was Ag-O (367.8 ± 0.4 eV). The presence of the Ag-O bond suggests that lignin is probably bound to the silver through oxygen [70]. A small amount of metallic silver Ag(0) was also determined on the Ag 3d orbital [71]. The Ag(B), C(B), and O(B) components resulted from different charging of the sample surface, leading to lower binding energy values compared with the other determined components. As a result, these components were not considered in the structural analysis of Lig-AgNPs.

**Figure 8:**
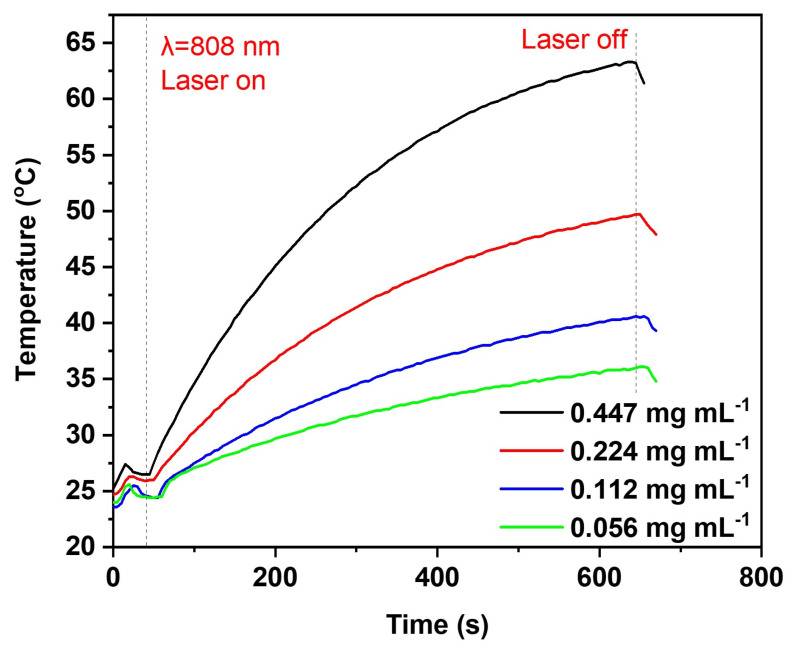
The temperature increase over time while irradiating Lig-AgNPs aqueous dispersions of various concentrations with 808 nm laser light at an intensity of 3.5 W cm^−2^.

As a final step of Lig-AgNPs characterization, their photothermal effect was examined over increasing concentration. The temperature increases over time attained by Lig-AgNPs dispersions at various concentrations during laser irradiation (3.5 W cm^−2^, λ = 808 nm), as measured by a thermocouple, are shown in Figure 8. A progressive temperature increase was observed for 10 min. When the laser was turned off (after ~10 min), the dispersions started to cool, demonstrating the control of the plasmonic photothermal properties of the hybrid Lig-AgNPs. It was observed that the temperature increase was concentration-dependent, with the highest temperature increase being 38.8 °C at a concentration of 0.447 mg mL^−1^.

To summarize, the comprehensive characterization of both the core and surface of isolated Lig-AgNPs reveals promising potential for other valuable applications, including within the biomedical field. The core, composed entirely of a pure metallic silver (Ag^0^), offers advantage compared with certain other green synthesis methods that may introduce AgCl [72]. This contamination can adversely affect the unique absorption properties of AgNPs due to the absence of associated localized surface plasmon resonance in AgClNPs [73], which is essential for specific intended applications of AgNPs [74,75,76]. Additionally, AgCl particles exhibit photosensitivity [77], leading to the oxidation of organic compounds upon irradiation and the generation of electrons that produce reactive oxygen radicals [78], potentially impacting their biocompatibility and toxicity. The incorporation of a biodegradable lignin envelope, confirmed by FTIR, XPS, and visual observation, enhances colloidal stability and is expected to mitigate cytotoxicity and environmental impact associated with widely used AgNPs [79,80,81]. The envelope could further support stabilizing the silver core to prevent Ag^+^ release contributing to cytotoxicity and ecotoxicity of AgNPs [82]. Moreover, the presence of lignin facilitates non-covalent interactions between AgNPs and cell membranes, which are crucial for potential biomedical applications [79,83,84,85]. Targeting, a critical aspect of these applications, could be achieved by efficient conversion of light into heat via plasmonic photothermal properties, enabling the use of AgNPs as photothermal agents [86,87].

## 3. Materials and Methods

### 3.1. Lignin Isolation

Lignin was isolated from beech sawdust (Lignocel HBS 150/500, Rettenmaier Sweden KB JRS, Helsingborg, Sweden) via organosolv fractionation as previously described [88]. Specifically, beech sawdust was treated in an air-heated multidigester (Haato, Vantaa, Finland) in 60% *v*/*v* ethanol in water solution of a solid-to-liquid ratio of 1/10 g mL^−1^ with the addition of 1% *w*/*w* wsulfuric acid calculated per dry biomass basis. Treatment took place at 180 °C for 60 min. After the organosolv, the pretreated solids (containing mainly the cellulose) were removed from the slurry via vacuum filtration, and the filtrate was processed through a rotary evaporator to remove the ethanol, rendering lignin insoluble to the aqueous solution. Finally, lignin was recovered by centrifugation at 10,000× *g* for 15 min at 4 °C (5804R; Eppendorf, Hamburg, Germany), freeze-dried (Lyoquest; Telstar, Terrassa, Spain), and stored at room temperature until further use.

### 3.2. Lignin Dissolution

Lignin was initially mixed with either ultrapure water (UPW) or a 60% aqueous ethanol solution (*v*/*v*) at varying initial concentrations of 1.0 g L^−1^, 2.5 g L^−1^, 5.0 g L^−1^, 10.0 g L^−1^, and 20.0 g L^−1^. Subsequently, a continuous addition of 1 M–10 M NaOH solution was added to the prepared mixture under constant stirring for 24 h. The pH was monitored until it stabilized at the desire value (10.00 ± 0.05; 11.00 ± 0.05, 12.00 ± 0,05). Insoluble lignin residues were separated from the mixture through vacuum filtration using a mixed cellulose ester filter with a pore site of 0.22 µm (Merck Millipore, IRL, Burlington, MA, USA). The concentration of the lignin solution was determined gravimetrically by measuring the content in the retentate. The prepared lignin solutions were immediately utilized for the synthesis of lignin-silver hybrid NPs (Lig-AgNPs).

### 3.3. Preparation and Separation of Lig-AgNPs

The synthesis of Lig-AgNPs involved the reaction between dissolved lignin and AgNO_3_ (VWR, USA) in UPW solution using various ratios. Initially, for primary screening, the reactions were conducted in a microtitrate plate P (GAMA GROUP a.s., České Budějovice, Czech republic) with a total volume of 210 µL (200 µL lignin + 10 µL AgNO_3_). In the subsequent scale-up phase, reaction with total volumes of 8.4 mL and 84.0 mL were performed in glass vessels (Scheme 1). The primary variable in the reactions was the silver ions–lignin mass ratio (m_Ag+_:lignin). The other reaction parameters, such as room temperature, 320 rpm, and a reaction time of 60 min, remained constant across all volumes.

Throughout the study, three fractions (P+S; P; S) were distinguished and subjected to analysis based on the methodology or scale-up phase (Scheme 1). Specifically, these fractions include P+S representing Lig-AgNPs characterized without subsequent separation, P representing the separated Lig-AgNPs, and S representing the supernatant depleted of Lig-AgNPs following our separation process. The separation process of NPs was carried out for samples prepared in total volumes of 8.4 mL and 84.0 mL. To maximize the isolation efficiency of the Lig-AgNPs from the unreacted components, a two-step centrifugation process was employed (49,054× *g*; 10 °C; 30 min), followed by resuspension of the pellets in ultrapure water (UPW) to the original sample volume. For characterization purposes, the separated nanoparticles were washed repeatedly (three times) with UPW. The prepared NPs were stored at 4 °C, either in the form of a colloidal suspension or lyophilized using a Heteo PowerDry LL 3000 Freeze Dryer (Thermo Electron Corporation, Waltham, MA, USA).

### 3.4. Characterization of Lig-AgNPs by UV-Vis

UV-Vis spectroscopy was employed to characterize Lig-AgNPs. The NPs were first diluted 40×–160× depending on the input concentration of the AgNO_3_ precursor. The choice of dilution solution, either UPW or 60% (*v*/*v*) aqueous ethanol solution, was based on the type of lignin solution used for the synthesis of Lig-AgNPs (UPW or 60% aqueous solution). The UV-Vis absorption spectrum was then scanned in the range of 300 nm to 800 nm, with a 5 nm step, using a Reader Infinite M900Pro (TECAN MTP, Männedorf, Switzerland).

### 3.5. Determination of Silver Mass Concentration by AAS

The mass concentration of silver in Lig-AgNPs samples was determined using an Agilent 280FS AA atomic absorption spectrometer (Agilent, Santa Clara, CA, USA). The concentration was assessed in three forms: unseparated (P+S) NPs, separated (P) NPs, and the supernatant (S).

To initiate the analysis, 200 µL of the sample was taken, and a double volume of 4% HNO_3_ (*v*/*v*) was added to it. The resulting mixture was then diluted with UPW to a final volume of 10 mL. This prepared solution was then subjected to analysis using the atomic absorption spectrometer to quantify the mass concentration of silver.

### 3.6. DLS Analysis and Zeta Potential Determination

The hydrodynamic diameter, polydispersity index (PDI), and zeta potential were measured using a Zetasizer Pro instrument (Malvern Panalytica, Malvern, UK). The obtained data were processed using ZS XPLORER software v.2.3.0.62 (Malvern Panalytical, Malvern, UK). For the analysis, the separated NPs samples were diluted with UPW to achieve an A_MAX (400–800) nm_ = 1.0 before measurement.

### 3.7. TEM Analysis

To investigate the size distribution and morphology of Lig-AgNPs, the air-dried solution of nanoparticles was visualized using TEM (EFTEM Jeol 2200 FS, JEOL, Tokyo, Japan) at an electron beam energy of 200 kV. Captured images were manually processed using ImageJ 1.53e open-source software, Java 1.8.0_172. For each nanoparticle, measurements of the major (2a) and minor (2b) axes were taken, allowing us to calculate the area (A) of the ellipse (Equation (1)). This calculated area was then compared with the area determined using a freehand drawing tool. The mean value of the particle diameter was subsequently determined according to Equation (2). Circularity (c) was calculated as the ratio of the particle area (A) to the area of a circle with equal perimeter $\left(p\right)$ (Equation (3)) [89]. The perimeter of the particle (p) was approximated according to Equation (4). This entire measurement process was repeated for 100 NPs in three separate repetitions.

(1) A=πab

(2) $$\overset{-}{d}=\frac{2a+2b}{2}$$

(3) $$c=\frac{4\pi A}{p^{2}}$$

(4) $$p\;\approx \;\pi \sqrt{2(a^{2}+b^{2}})$$

### 3.8. Lignin and Lig-AgNPs Characterization by FTIR 

The FTIR spectra of the lyophilized samples were acquired using a Nicolet 6700 FTIR spectrometer (Thermo Fisher Scientific, Waltham, MA, USA) coupled with the diamond ATR GladiATR (PIKE, Madison, WI, USA) and DTGS KBr detector. The spectral analysis was conducted in the range of 4000–400 cm^−1^ with a resolution of 4 cm^−1^, utilizing 64 accumulations of spectra and Happ-Genzel apodization. To correct the baseline, air humidity and CO_2_ signal signals were subtracted from the spectra.

### 3.9. Characterization of Lig-AgNPs by XRD

The crystal structure and crystallite size of lyophilized Lig-AgNPs samples were determined using X-ray diffraction (XRD) with a PANalytical X’Pert PRO instrument (PANanalytical, Almelo, The Netherlands). Powder diffraction data were collected at room temperature using an X’Pert^3^ θ-θ powder diffractometer with Bragg–Brentan parafocusing geometry and CuK_α_ radiation (λ = 1.54060 Å, U = 40 kV, I = 30 mA).

The data were scanned over an angular range of 5°–90°(2θ) with a step size of 0.0390° (2θ) and a counting time of 354.96 s step^−1^. The ultra-fast 1D detector PIXCEL was employed for data collection. The data evaluation was carried out using HighScore Plus 4.0 software (Malvern Panalytical, Malvern, UK) and the PDF-4+ database as a reference.

### 3.10. Characterization of LigNPs by XPS

X-ray photoelectron spectroscopy (XPS) measurements were conducted using an ESCA Probe P spectrometer (Omicron Nanotechnology GmbH, Taunusstein, Germany) equipped with a monochromatic Al K_α_ X-ray source (hν = 1486.7 eV). The measurements were performed at a pressure of approximately 10^−8^ Pa. To compensate for charging effects, a low-energy electron flood gun (SL 1000, Omicron Nanotechnology GmbH, London, UK) was employed.

The Lig-AgNPs suspensions were deposited onto indium carriers to create a film for analysis. Wide XPS spectra and core levels were recorded with a step size of 0.4 eV and 0.1 eV, respectively. The electron binding energies of the different core levels were corrected according to the C-C (sp2, sp3) bonds of the C1s peak at 284.8 eV. Data evaluation and analysis of the spectra were performed using Casa XPS 2.3.26 software.

### 3.11. Photothermal Effect

The photothermal effect of the Lig-AgNPs was explored under laser irradiation at 808 nm from a fiber-coupled diode laser with a top-hat diffuser with a square output profile (Laser Century). The temperature increase was monitored using a thermocouple. The laser power after the diffuser was measured using a Thorlabs S425C thermal optical power meter. The diffuser height was adjusted to achieve the desired beam intensity (3.5 W cm^−2^).

## 4. Conclusions

In conclusion, our study shows the advantageous use of the waste substrate lignin for the formation of Lig-AgNPs through careful control of the reaction conditions. By understanding the reaction mechanism governing the synthesis of AgNPs and the solubility of lignin, we strategically selected pH 11 and water conditions over a 60% ethanol aqueous solution. This was also supported by the linear regression of the maxima of the absorption spectra of Lig-AgNPs, indicating that the conversion of the silver precursor to silver nanoparticles occurs more efficiently in water.

For our scale-up process, we identified a lignin input concentration of 5 g L^−1^ as suitable, achieving high conversion of the silver precursor to nanoparticles (0.013 mol L^−1^ of Ag) at a high m_Ag_:m_Lig_ reaction ratio, thereby maximizing lignin’s role as a reducing and capping agent. Additionally, a concentration of 10 g L^−1^ provided a high yield per volume (0.018 mol L^−1^ of Ag) while maintaining the absorption signal of Lig-AgNPs, facilitating efficient production without the need to increase the volume of the reaction. The use of AAS, DLS, and TEM confirmed the suitability of using UV-Vis spectroscopy as a simple and rapid method for determining the appropriate reaction conditions for the surface plasmon resonance response of AgNPs in small volumes. The process proposed by us verified the suitability of the conditions for the formation of homogeneous Lig-AgNPs, even during scale up, while maximizing the utilization of the silver precursor. Furthermore, our results indicate that maintaining an appropriate reaction ratio is crucial for achieving suitable conditions in the synthesis of Lig-AgNPs, while the input concentration of lignin appears to be a secondary factor. A detailed analysis via XRD and XPS revealed a pure face-centered cubic structure of metallic silver, with lignin being present through an oxygen-mediated bond. Electron microscopy confirmed the presence of lignin on the surface and FTIR revealed that, in addition to its reducing groups, the packing of lignin and its ability to stabilize an oxidized state are also important factors in the formation of AgNPs.

Overall, our study presents a robust synthesis method for Lig-AgNPs, addressing key limitations of other green synthesis approaches and offering potential industrial scalability. Our simple, room temperature process using abundant lignin holds promise for valuable applications. The lignin envelope could provide stability and an additional functional group for modification, expanding the possibilities for lignin use in various applications requiring a water environment.

## Figures and Tables

**Table 1:** Lignin solubility in NaOH aqueous solution.

Input Lig [g L^−1^]	pH 10		pH 11		pH 12	
	Lignin [g L^−1^]	Dissolution [%]	Lignin [g L^−1^]	Dissolution [%]	Lignin [g L^−1^]	Dissolution [%]
1.00	0.52 ± 0.03	52 ± 3	0.88 ± 0.01	88 ± 1	~1.00	~100.0
2.50	1.05 ± 0.07	42 ± 3	1.99 ± 0.02	80 ± 1	~2.50	~100.0
5.00	1.91 ± 0.12	38 ± 2	4.12 ± 0.14	82 ± 3	~5.00	~100.0
10.00	3.56 ± 0.37	36 ± 4	8.05 ± 0.26	80 ± 3	~10.00	~100.0
20.00	6.64 ± 0.12	33 ± 1	14.02 ± 0.71	70 ± 4	~20.00	~100.0

**Table 2:** Linear regression parameters y=k·x+q of the maximum absorbance values ranging from 400 nm to 800 nm of unseparated Lig-AgNPs (P+S) on the reaction mass ratio between Ag^+^ and lignin at different initial lignin concentrations for reactions performed in a total volume of 210 µL.

Reaction Medium	Initial Lignin Conc. [g L^−1^]	Slope (k)	Intercept (q)	R-Square (COD)
Water		1.047 ± 0.065	0.023 ± 0.019	0.967
	2.50	2.762 ± 0.151	0.078 ± 0.036	0.977
	5.00	5.965 ± 0.285	0.231 ± 0.051	0.984
	10.00	12.47 ± 1.171	0.436 ± 0.163	0.950
	20.00	17.595 ± 1.337	0.681 ± 0.214	0.967
60% (*v*/*v*) Ethanol	1.00	1.864 ± 0.161	0.026 ± 0.015	0.964
	2.50	3.883 ± 0.421	0.114 ± 0.032	0.955
	5.00	6.267 ± 0.565	0.285 ± 0.063	0.953
	10.00	9.857 ± 0.857	0.439 ± 0.079	0.964
	20.00	19.047 ± 2.307	0.841 ± 0.145	0.958

**Table 3:** Linear regression parameters y=k * x+q of the maximum absorbance values ranging from 400 nm to 800 nm of unseparated (P+S) and separated (P) Lig-AgNPs on the reaction mass ratio between Ag^+^ and lignin at different initial lignin concentrations for reactions performed in a total volume of 8.4 mL.

Reaction Medium	Initial Lignin Conc. [g L^−1^]	Slope (k)	Intercept (q)	R-Square (COD)
Water	5.00 (P+S)	6.767 ± 0.100	0.244 ± 0.023	0.998
	5.00 (P)	7.174 ± 0.069	0.016 ± 0.016	0.999
	10.00 (P+S)	12.153 ± 0.312	0.540 ± 0.057	0.995
	10.00 (P)	13.799 ± 0.168	0.044 ± 0.031	0.999

**Table 4:** Characteristics of Lig-AgNPs prepared in a total volume of 8.4 mL and 84.0 mL at two input lignin concentrations and mass reaction ratios m_Ag_:m_Lig_ 5.0 g L^−1^ 0.37 and 10.0 g L^−1^ 0.25.

Method	5.0 g L^−1^–0.37 m_Ag_:m_Lig_		10.0 g L^−1^–0.25 m_Ag_:m_Lig_	
	8.4 mL	84.0 mL	8.4 mL	84.0 mL
UV-Vis (P+S)	2.69 ± 0.07	2.76 ± 0.8	3.61 ± 0.16	3.77 ± 0.25
UV-Vis (P)	2.71 ± 0.05	2.67 ± 0.04	3.50 ± 0.05	3.45 ± 0.22
DLS-Z-Average [nm]	32.8 ± 3.4	36.2 ± 1.3	26.4 ± 1.6	27.4 ± 1.5
DLS-PDI	0.31 ± 0.05	0.40 ± 0.02	0.29 ± 0.05	0.32 ± 0.06
Zeta potential [mV]	−31.2 ± 5.6	−55.8 ± 2.1	−41.1 ± 1.9	−58.4 ± 1.9
TEM core median size [nm]	15 ± 3	13 ± 2	15 ± 2	12 ± 2
TEM core median circularity	0.991 ± 0.010	0.993 ± 0.016	0.990 ± 0.015	0.985 ± 0.018
AAS-Lig-AgNPs yield [%]	96.1 ± 4.0	96.7 ± 4.0	99.5 ± 4.0	98.9 ± 4.0

## Data Availability

Data are available upon reasonable request from the corresponding authors.

## Supplementary Materials

The following supporting information can be downloaded at: https://www.mdpi.com/article/10.3390/molecules29102360/s1, Figure S1: UV-Vis absorption spectra of  lignin;  unseparated (P+S) Lig-AgNPs;  separated (P) Lig-AgNPs;  supernatant (S). Figure S2: Samples of unseparated (P+S) Lignin–Ag-NPs prepared in a total volume of 8.4 mL, UPW, pH 11.00 ± 0.05. Mass reaction ratios m_Ag_:m_Lig_ increase from sample 1 to sample 12. (**a**) Initial lignin concentration of 5.0 g L^−1^; (**b**) initial lignin concentration of 10.0 g L^−1^. Figure S3: Microscopy revealing the presence of a lignin envelope surrounding the silver core of Lig-AgNPs: (**a**) transmission electron microscopy (TEM); (**b**) scanning electron microscopy (SEM).
