# Supplementary material for: Synthesis and Characterization of Lignin-Silver Nanoparticles
Source: Molecules. 2024 May 16;29(10):2360. doi: 10.3390/molecules29102360 (PMC11123738; doi:10.3390/molecules29102360)
Supplement: Supplementary file 1 [file molecules-29-02360-s001.zip › molecules-3008522-supplementary.pdf]

molecules

MDPI

Supplementary Materials

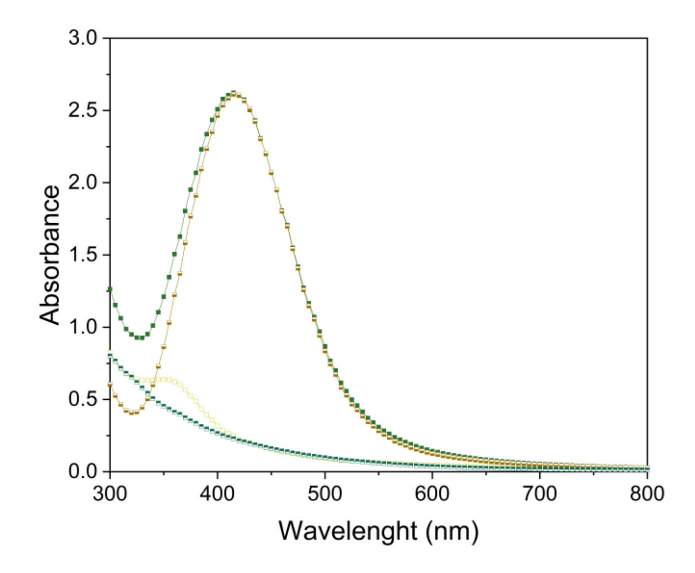

Figure S1. UV-Vis absorption spectra of □ lignin; ■ unseparated (P+S) Lig−AgNPs; ■ separated (P) Lig−AgNPs; ■ supernatant (S).

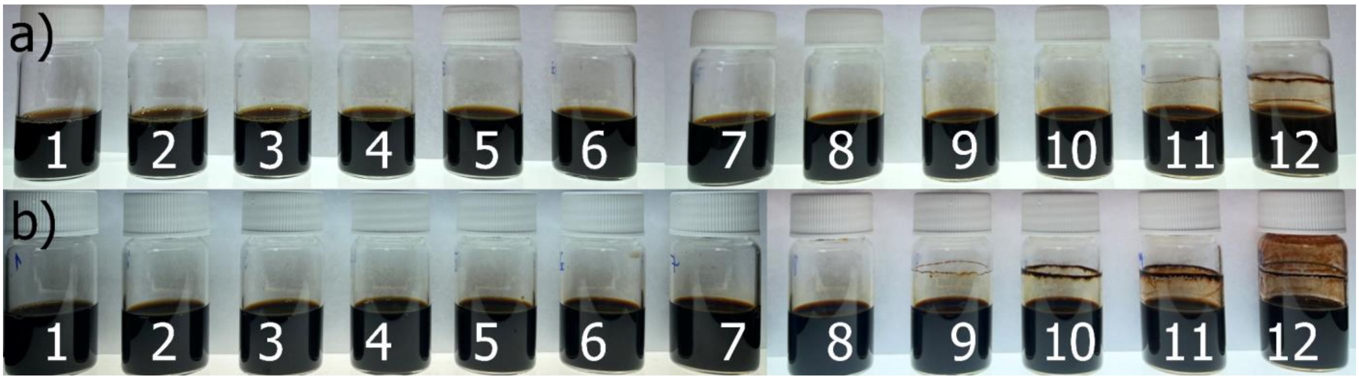

Figure S2. Samples of unseparated (P+S) Lig−AgNPs prepared in a total volume of 8.4 ml, UPW, pH 11.00 ± 0.05. Mass reaction ration m_{Ag}:m_{lig} increase from sample 1 to sample 12 (**a**) initial Lignin concentration 5.0 g L^{-1} (**b**) initial Lignin concentration 10.0 g L^{-1}.

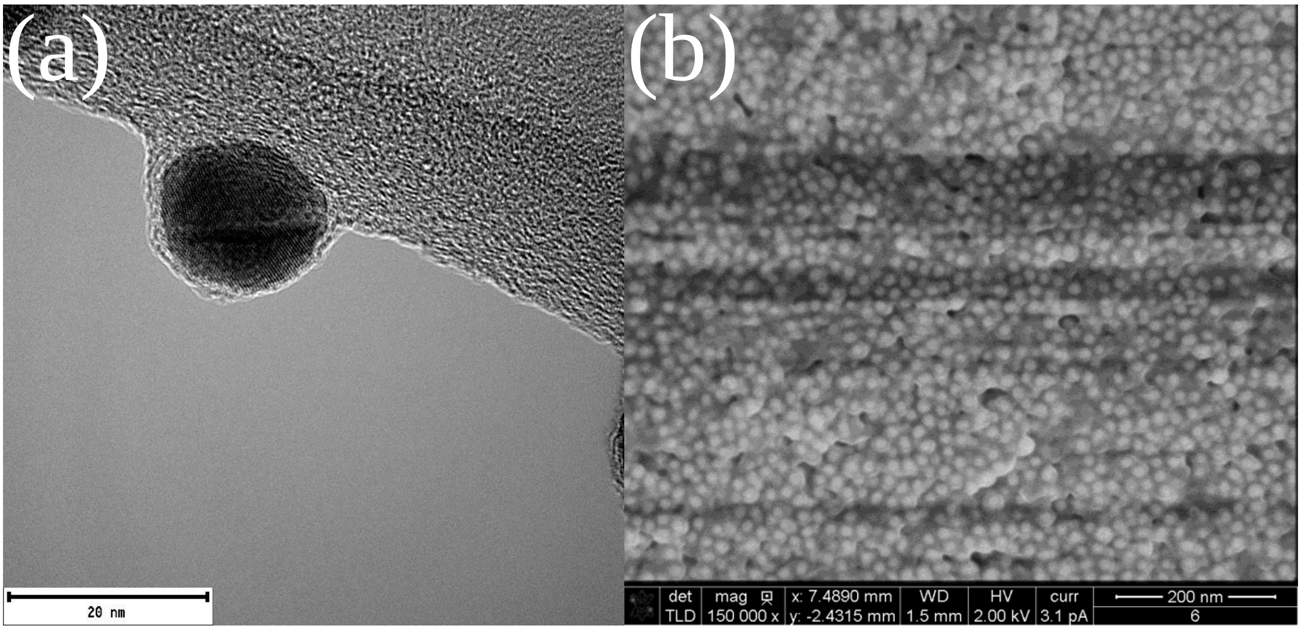

Figure S3. Microscopy revealing the presence of a lignin envelope surrounding the silver core of Lig−AgNPs: (**a**) transmission electron microscopy (TEM); (**b**) scanning electron microscopy (SEM).
